# Therapeutic Potential of Electromyostimulation (EMS) in Critically Ill Patients—A Systematic Review

**DOI:** 10.3389/fphys.2022.865437

**Published:** 2022-05-09

**Authors:** Maryam Balke, Marc Teschler, Hendrik Schäfer, Pantea Pape, Frank C. Mooren, Boris Schmitz

**Affiliations:** ^1^ St. Marien Hospital Cologne, Department of Early Rehabilitation, Cologne, Germany; ^2^ Department of Rehabilitation Sciences, Faculty of Health, University of Witten/Herdecke, Witten, Germany; ^3^ DRV Clinic Königsfeld, Center for Medical Rehabilitation, Ennepetal, Germany

**Keywords:** critical illness, ICU-acquired weakness, early rehabilitation, critical illness myopathy, critical illness polyneuropathy, electromyostimulation, neuromuscular electrical stimulation

## Abstract

Ample evidence exists that intensive care unit (ICU) treatment and invasive ventilation induce a transient or permanent decline in muscle mass and function. The functional deficit is often called ICU-acquired weakness with critical illness polyneuropathy (CIP) and/or myopathy (CIM) being the major underlying causes. Histopathological studies in ICU patients indicate loss of myosin filaments, muscle fiber necrosis, atrophy of both muscle fiber types as well as axonal degeneration. Besides medical prevention of risk factors such as sepsis, hyperglycemia and pneumonia, treatment is limited to early passive and active mobilization and one third of CIP/CIM patients discharged from ICU never regain their pre-hospitalization constitution. Electromyostimulation [EMS, also termed neuromuscular electrical stimulation (NMES)] is known to improve strength and function of healthy and already atrophied muscle, and may increase muscle blood flow and induce angiogenesis as well as beneficial systemic vascular adaptations. This systematic review aimed to investigate evidence from randomized controlled trails (RCTs) on the efficacy of EMS to improve the condition of critically ill patients treated on ICU. A systematic search of the literature was conducted using PubMed (Medline), CENTRAL (including Embase and CINAHL), and Google Scholar. Out of 1,917 identified records, 26 articles (1,312 patients) fulfilled the eligibility criteria of investigating at least one functional measure including muscle function, functional independence, or weaning outcomes using a RCT design in critically ill ICU patients. A qualitative approach was used, and results were structured by 1) stimulated muscles/muscle area (quadriceps muscle only; two to four leg muscle groups; legs and arms; chest and abdomen) and 2) treatment duration (≤10 days, >10 days). Stimulation parameters (impulse frequency, pulse width, intensity, duty cycle) were also collected and the net EMS treatment time was calculated. A high grade of heterogeneity between studies was detected with major cofactors being the analyzed patient group and selected outcome variable. The overall efficacy of EMS was inconclusive and neither treatment duration, stimulation site or net EMS treatment time had clear effects on study outcomes. Based on our findings, we provide practical recommendations and suggestions for future studies investigating the therapeutic efficacy of EMS in critically ill patients.

**Systematic Review Registration**: [https://www.crd.york.ac.uk/prospero/], identifier [CRD42021262287].

## Introduction

Critical illness requires intensive care and may lead to invasive or non-invasive mechanical ventilation. Frequent complications are metabolic disturbances, multiple medication, malnutrition, as well as sepsis, which most likely constitutes the predominant negative factor for further convalescence (Friedrich et al., 2015; Hashem et al., 2016). ICU treatment may become necessary for several reasons including chronic obstructive pulmonary disease (COPD) and other conditions for respiratory failure, cardiac arrest and/or cardiac surgery, abdominal surgery, infectious diseases, septic shock, and neurologic disorders. Recently, severe COVID-19 pneumonia caused by SARS-CoV-2 infection has added to this list. ICU treatment may cause several short- and long-term complications including physical (especially pulmonary and musculoskeletal), cognitive, and mental decline, affecting not only survivors but also relatives and caregivers (Needham et al., 2012; Govindan et al., 2014; Brunkhorst et al., 2020; Rousseau et al., 2021).

### Intensive Care Unit-Acquired Weakness

Among the common short- and long-term complications, ICU-acquired weakness affects about 40% of all critically ill patients and up to 100% of patients with septic shock or severe sepsis with organ failure (Latronico et al., 2012; Appleton et al., 2015). ICU-acquired weakness is a disease entity including critical illness polyneuropathy (CIP), critical illness myopathy (CIM), disuse atrophy, sepsis-induced myopathy, and steroid-denervation (Friedrich et al., 2015). There are fluent transitions between these conditions and the often long-term disabling condition of CIP, a large fiber neuropathy, and CIM, a primary myopathy, which can only be objectified by electrophysiological examination and/or biopsy studies (Latronico et al., 2012; Latronico et al., 2017). In addition to CIP and CIM, involvement of the autonomous nervous system and small fibers may contribute to ICU-acquired weakness (Hermans and Van den Berghe, 2015). Since neurophysiological studies are rarely possible on ICU, CIP or CIM are usually diagnosed based on clinical presentation with generalized, symmetrical, flaccid paresis of limb muscles sparing facial and ocular muscles. Muscle strength is usually categorized by manual muscle strength testing using the Medical Resource Council (MRC) sum-score, with a total MRC sum-score <48 indicating ICU-acquired weakness (Fan et al., 2014). Of note, the MRC score at ICU discharge is associated with 5-years mortality and MRC is thus often used to assess the success of early rehabilitation programs (De Jonghe et al., 2002; Fan et al., 2014; Van Aerde et al., 2021).

With regard to the underlying mechanisms of ICU-acquired weakness, muscle degradation with proteolysis has been accounted for a rapid loss of muscle mass with up to 20% already in the first 10 days of ICU stay (Puthucheary et al., 2013; Lad et al., 2020). On the cellular level, a reception of myocyte cross-sectional area has been detected already 5 days after ICU admission, with an estimated loss of fiber thickness of 3–4% per day (Helliwell et al., 1998). This process appears to be accompanied by a loss of myosin filaments and myosin-associated proteins, potentially based on decreased expression of different myosin heavy chain (MyHC) transcripts and increased MyHC protein degradation by early activation of atrophy-related genes (Larsson et al., 2000; Wollersheim et al., 2014; Heras et al., 2019). Early induction of the ubiquitin–proteasome system and activation of the inflammatory nuclear factor kappa B (NFκB) involved in critical illness muscle atrophy and muscle wasting has also been described (Cai et al., 2004; Schefold et al., 2020) while muscle protein synthesis has been suggested to be downregulated in CIM (Weber-Carstens et al., 2013). In electrophysiological studies, a large number of ICU patients showed no response of muscle membranes to direct muscle stimulation already after 1 week of ICU treatment, which was associated with smaller type II muscle fiber cross-sectional area (Bierbrauer et al., 2012). Moreover, typical skeletal muscle striation may be lost in ICU patients and sarcomere disruption has been described in CIM patients 1 week after ICU discharge (Larsson et al., 2000; Dos Santos et al., 2016). Analyses of vastus lateralis satellite cell content 7 days and 6 months after ICU discharge suggested lower satellite cell content in patients with sustained muscle wasting at 6 months follow-up. Of note, muscle vascularization in terms of capillary-to-myofiber ratio correlated with the satellite cell content (Dos Santos et al., 2016). To this respect, sepsis, a major cause and complicating factor of critical illness, induces elevation of pro-inflammatory cytokines and metabolic imbalances that induced long-term impairment of satellite cells and inefficient muscle regeneration. In animal experiments, delivery of mesenchymal stem cells has been shown to restore mitochondrial and metabolic function in satellite cells, and improving muscle strength (Rocheteau et al., 2015). This is of relevance, since a reduction in mitochondrial content as well as signs of mitochondrial dysfunction have been observed in critical illness (Wollersheim et al., 2014; Jiroutkova et al., 2015). Of note, the extent of mitochondrial functional impairment correlates with disease severity and the ability of early mitochondrial biogenesis activation has been linked to survival of critical illness (Brealey et al., 2002; Carre et al., 2010).

To reduce or prevent ICU-acquired weakness, (very) early rehabilitation is necessary and widely implemented as an ICU standard treatment from day one until dischargement and continued in the post-ICU and outpatient setting (Needham et al., 2012). Early rehabilitation therapy has been proven to be safe and feasible with well-established mobilization protocols (Jang et al., 2019). Depending on the level of consciousness and muscular abilities, protocols start with passive range of motion therapy, followed by more advanced mobility therapy consisting of active resistance physical therapy and subsequent transfer to sitting position in bed/chair, standing, walking, and manual functioning exercises (Morris et al., 2008). Early rehabilitation has been proven to be effective to increase patients’ independence in activities of daily living and ambulation on hospital discharge (Burtin et al., 2009; Schweickert et al., 2009).

### Electromyostimulation

Since most ICU patients are still unconscious during the first days of ICU therapy, early rehabilitation teams have tried to establish strategies to activate muscular function even in this state. One of these methods is Electromyostimulation [EMS, also termed neuromuscular electrical stimulation (NMES)] (Gerovasili et al., 2009), which includes the application of local surface electrodes over the bellies or motor points of one (or multiple) selected larger superficial muscles, aiming to stimulate neuromuscular activity with tangible and visible muscle actions (Takeda et al., 2017). EMS can be delivered passively to an inactive muscle, or actively by stimulation during a voluntary muscle activity, or by means of Functional Electrical Stimulation (FES), that applies electric stimulation to a paralyzed muscle during certain movements like walking or grasping, and which can be combined with task-specific therapy (Furlan et al., 2021). A more recent development is Whole-Body EMS (WB-EMS), which addresses larger muscle areas including abdomen, chest, and back muscles, as well as gluteal, leg and arm muscles allowing simultaneous stimulation of all parts of the body (Teschler and Mooren, 2019). EMS induces the non-selective recruitment of all muscle fibers (both types I and II) in the electrical field and has been shown to enhance muscle mass and function in athletes and healthy adults (Gregory and Bickel, 2005; Vanderthommen and Duchateau, 2007; Kemmler et al., 2021). Of note, EMS may also exert long-term effects even if stimulation is discontinued as muscle strength and cross-sectional area (CSA) have been reported to decrease after 4 weeks of detraining, but not to the pretraining level (Gondin et al., 2006). If used properly, WB-EMS seems to be a safe training option also in healthy subjects unable to join conventional training programs (Kemmler et al., 2018) and may be used to enhance muscle mass in the elderly in risk of sarcopenia and its complications (Kemmler and von Stengel, 2013; Kemmler et al., 2016; Teschler et al., 2021). With regard to the clinical application of EMS to reduce the effects of immobility, evidence for its efficacy comes from studies investigating patients with immobilized limbs due to surgery. In this population, EMS has been shown to prevent muscle strength loss of the quadriceps femoris and to induce faster return to pre-surgery levels (Paillard et al., 2005). Comparable effects have been reported after hip fractures also in elderly women (Snyder-Mackler et al., 1991; Lamb et al., 2002) and clinical populations including COPD (Hill et al., 2018), stroke (Stein et al., 2015), and spinal cord injury (Sheffler and Chae, 2007), certain oncologic conditions (O'Connor et al., 2021), heart failure (Ploesteanu et al., 2018), and advanced progressive disease in general (Jones et al., 2016). However, the combination of EMS with voluntary muscle contraction seems to be more effective and the effects of passive stimulation appear less clear (Dehail et al., 2008), which may also affect the application of EMS on ICU. While optimal parameters including mode of current application, session duration, intensity and application frequency have been investigated intensively in athletes and healthy individuals (Filipovic et al., 2011), consensus for (WB-)EMS application in the ICU setting has not been reached. Results from recent systemic reviews and meta-analyses appear inconsistent regarding the efficacy of EMS in critically ill patients and the optimal EMS application and treatment conditions remain to be identified (Edwards et al., 2014; Burke et al., 2016; Zayed et al., 2020).

## Objective

This work aimed to systematically review the therapeutic evidence on EMS applications in critical illness on ICUs and to provide a qualitative summary of results based on main modifiers. Based on these findings, information on optimal EMS application and treatment conditions could be identified and recommendations for future clinical studies on ICU-based EMS treatment of critically ill patients will be developed.

## Methods

### Study Design and Eligibility Criteria

We performed a systematic review (CRD42021262287) in accordance with the PRISMA guidelines and following the suggestions for reporting on qualitative summaries (Lucas et al., 2007; Campbell et al., 2020). Any original article reporting on EMS was considered for the analysis. Studies had to report on EMS (or comparable therapy, see definitions) in critically ill patients treated on ICU, on the specific stimulation devices and stimulation parameters, and have at least one functional outcome measure such as muscle function, functional independence, or weaning outcomes. Only articles available as full text (after an attempt to contact the corresponding author), and reporting on patients aged >18 years were included. Articles were not eligible if they 1) included healthy subjects, 2) focused on other conditions but critical illness, 3) did not include humans, or 4) were no original research (review, book, or conference abstract). Articles were excluded if they 1) focused on histological and morphological changes only, 2) investigated the acute effects of EMS (i. e. after a single EMS session), 3) were not written in English (full text), 4) were grey literature or website articles, 5) did not clearly report on included subjects, type of intervention or applied treatment, outcome measures, and statistical analysis, or 6) were no randomized controlled trials (RCTs).

### Search Strategy and Data Sources

**FIGURE 1:**
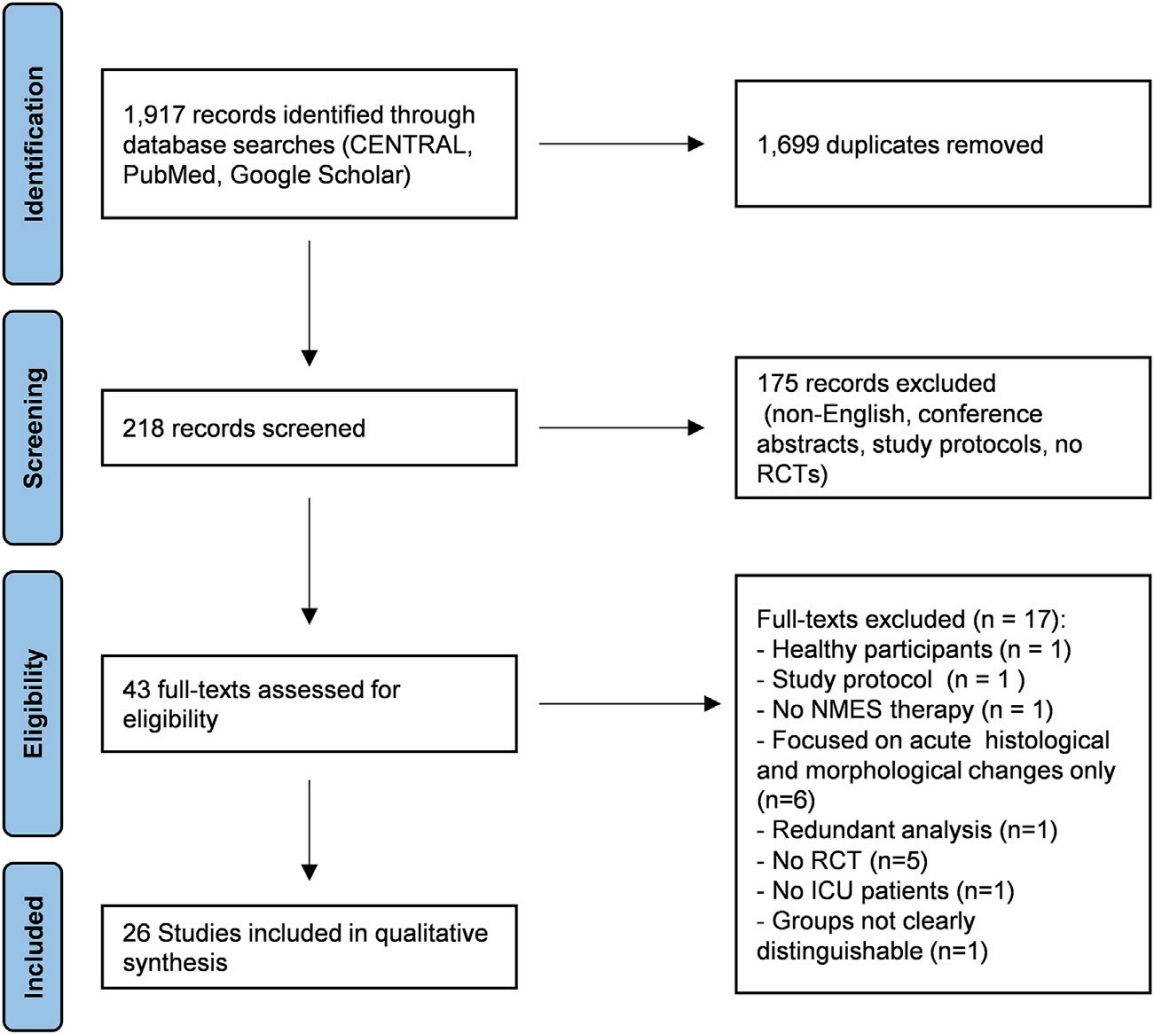
PRISMA flow chart. Out of 1,917 records identified through database searches, 43 full texts were assessed for eligibility and 26 studies were included in the qualitative analysis. Eligible articles had to report on electromyostimulation (EMS) or neuromuscular electrical stimulation (NMES) in critically ill patients treated on intensive care unit (ICU) in a randomized controlled trial (RCT) with at least one functional outcome (muscle function, functional independence, weaning outcomes).

A systematic search of the literature was conducted using PubMed (Medline), CENTRAL (including Embase, CINHAL), and Google Scholar for articles published until July 2021. A sensitive search strategy was developed, which combined variations and combinations of the following Medical Subject Headings and keywords: “critical illness”, “intensive care”, “muscle weakness”, “critical illness myopathy”, “critical illness polyneuropathy”, “ICU-acquired weakness”, “neuromuscular electrical stimulation”, “electromyostimulation”, “electric muscle stimulation”, “EMS”, “NMES”, “CIP”, “CIM”, “critical illness AND rehabilitation”, “critical illness AND therapy”, “critical illness AND treatment”, “transcutaneous electric nerve stimulation”, “TENS”, and “electric stimulation therapy”. Manual searches were also performed using reference lists from identified articles and reviews. The steps of report identification, screening and processing are documented in the PRISMA flow-chart (Figure 1). Fulfilment of eligibility criteria were discussed if unclear (MB and BS) until consensus was reached and upon disagreement, a third person was consulted to determine inclusion.

### Study Selection, Data Extraction and Aggregation

Data were extracted by two reviewers (MB and BS) and tables were created including information on first author, year of publication, number, age and sex of patients included, type of intervention, underlying diseases and incidence of sepsis, disease severity according to SOFA (Sepsis Related Organ Failure Assessment) and/or APACHE (Acute Physiology And Chronic Health Evaluation) score, primary and secondary outcomes, stimulation sites/muscle groups and duration, type of device, manufacturer, and stimulation parameters. In case of imprecise, uncommon, unclear/conflicting, or missing descriptions of used method, test participants, or stimulation parameters, full texts and manufacturer resources (device manual, website) were screened (MB, MT and BS) for additional information or authors were contacted to complete necessary information. To provide overall means for age, treatment duration, and clinical scores from study subgroups, median and range were converted to mean and SD (Zeng et al., 2020) and global (weighted) mean and SD was then calculated as described (Bland and Kerry, 1998; Schmitz et al., 2018).

### Grouping of Studies and Synthesis

To provide a structured qualitative summary, studies were grouped by main modifiers “stimulated muscles/muscle area” and “treatment duration”. In terms of treatment duration, two main groups of studies were identified. Studies either applied treatment for ≤10 days, or applied treatment for >10 days (up to 28 days). Heterogeneity was investigated using ordering tables including the respective main outcome [muscular and functional outcome measures, disease severity (SOFA/APACHE), biomarkers, or weaning outcomes] and the above-mentioned modifiers. Certainty of the evidence was addressed using an evaluation of how directly the included studies address the planned question/applied methodology (measurement validity), the number of studies and participants in each group, the consistency of effects across studies, and the risk of bias of the studies.

### Definitions

Only studies that used non-implanted electromyostimulation (EMS) devices - also called neuromuscular electrostimulation (NMES) - were included in this review. Both forms of EMS delivery, passive application, or active application in combination with voluntary muscle contraction, were eligible. The latter is frequently also referred to as functional electrostimulation (FES) and studies reporting on FES were included if comparable devices and stimulation parameters were used defined as follows. Devices for EMS therapy had to comply with the following parameters: monophasic, biphasic or polyphasic waveform of 20–100 Hz frequency, pulse width >100 µs, rectangular or sine waveform, and current intensity (mA) capable of producing visible muscle contractions (Takeda et al., 2017). In case “ms” was used for microsecond, the abbreviation was uniformly converted to “µs”. Critical illness was defined as a life-threatening condition with the necessity to replace the function of multiple organ systems including mechanical ventilation and intensive monitoring on a specialized ward [Intensive Care Unit (ICU)] (Kelly et al., 2014).

### Calculation of the Applied Net Electromyostimulation Treatment Time

To estimate the overall net EMS treatment time in the individual studies, the number of treatment days was extracted or calculated. If days of EMS treatment were not reported explicitly, the number of planned treatment days or the given length of ICU stay was used. Total treatment duration was then calculated using the treatment frequency (sessions per day/week) and session duration. Since the applied duty cycle determines the duration of stimulation and relief periods per interval and thus the actual time (seconds) an electrical current is applied to the muscle per minute, duty cycle information was then used to calculate the overall applied net EMS treatment time in minutes. No further calculations were conducted since the current (mA) necessary to induce muscle contractions is affected by individual factors including local body fat, skin thickness, and muscle mass and most studies reported using visible muscle contraction instead of predefined currents as application principle. Calculation of the recommended EMS parameters was performed based on a sufficiently large number of studies reporting significant improvement of muscle outcomes after stimulation of leg (and arm or abdomen) muscles according to Filipovic et al. (2011) using overall study means.

### Quality Assessment

The methodological quality of the studies was assessed using the 11-item PEDro scale based on the Delphi list developed by Verhagen and colleagues (Verhagen et al., 1998). For our analysis, we determined (in accordance with the above-mentioned eligibility criteria) that the following item had to be scored “yes”: subjects were randomly allocated to groups or, for intraindividual right-left comparison, extremities were randomly assigned to treatment or control. Participants who were unconscious throughout the whole course of EMS treatment were rated as blinded. All other items were rated by to reviewers (MB and BS) to determine the level of bias in each study. Disagreements were resolved by discussion if necessary. The researchers were not blinded to study authors, results, or publication journal.

## Results

Out of 1,917 identified records, 26 studies fulfilled the eligibility criteria and were included in the quantitative analysis (Figure 1). Studies were grouped according to two main modifiers “stimulated muscles groups” and “treatment duration” (Table 1). The stimulated muscle groups reported were “quadriceps muscle only”, “two to four leg muscle groups”, “legs and arms”, “abdomen in combination with two to four leg muscles”, and “chest and abdomen”. Since the observed median treatment duration was 10 (4–28) days, treatment duration was grouped by “treatment ≤10 days” and “treatment >10 days”. Seven studies (27%) used an intraindividual control, comparing the stimulated side of the body to non-stimulated regions (equal or different muscle groups). All other studies used treatment and control groups for comparison. Devices and respective stimulation parameters as well as treatment duration and the calculated net EMS treatment time (minutes) are presented in Table 2.

**TABLE 1:** Summary of references.

References	Sample (n)	Age (years)	Session duration (min.)	Baseline SOFA score	Baseline APACHE II score	Diagnosis	Main outcome	Influence on				
								Muscle strength/volume/histology	SOFA/APACHE II score	Functional independence/ambulation	Biomarker	Duration of mechanical ventilation/ICU length of stay
**Stimulated muscle: quadriceps muscle only**												
**Treatment ≤ 10d**												
Chen et al. (2019)	33 (SG = 16, CTRL = 17)	75.7 ± 16.1	60 min	n.a	20.5 ± 6.8	Respira-tory failure	Increase in leg muscle strength	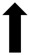	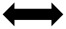	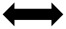	n.a	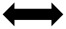
Dirks (2015)	6 (SG = 6, CTRL* = 6)	63.0 ± 6.0	60 min	n.a	29.3 ± 3.7	Multiple	Decrease in type 1 and type 2 muscle–fiber CSA in CTRL leg, no muscle atrophy in stimulated leg	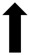	n.a	n.a	n.a	n.a
Fischer et al. (2016)	54 (SG = 27, CTRL = 27)	66.5 ± 14.6	60 min	7.3 ± 9.4	n.a	Cardiac surgery^#^	Increased muscle strength, no difference in muscle layer thickness	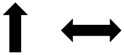	n.a	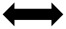	n.a	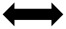
Koutsioumpa et al. (2018)	80 (SG = 38, CTRL = 42)	65.1 ± 12.7	60 min	7.5 ± 4.2	19.1 ± 8.0	Multiple^#^	No effect on myopathy, increase in MRC	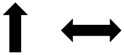	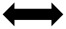	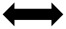	n.a	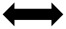
Poulsen et al. (2011)	8 (SG = 8, CTRL* = 8)	67.7 ± 7.0	60 min	11.4 ± 4.4	24.6 ± 7.9	Septic shock	No difference in muscle volume between stimulated and non-stimulated thigh	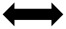	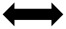	n.a	n.a	n.a
Strasser et al. (2009)	26 (SG = 26, CTRL* = 26)	60.0 ± 10.0	30 min	n.a	n.a	Abdominal surgery	Increase in total RNA content and reduced protein degradation	n.a	n.a	n.a	Increased total RNA content	n.a
**Treatment > 10d**												
Abu-Khaber et al. (2013)	80 (SG = 40, CTRL = 40)	58.3 ± 6.1	60 min	n.a	25.3 ± 6.1	Respira-tory failure^#^	Reduced MV time	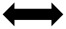	n.a	n.a	n.a	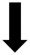
Berney et al. (2020) ^‡^	162 (SG = 80, CTRL = 82)	59.1 ± 14.0	60 min	10.4 ± 4.9	15.9 ± 8.7	Multiple^#^	No effect on muscle strength	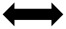	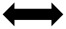	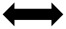	n.a	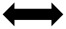
Fossat et al. (2018) ^‡^	312 (SG = 158, CTRL = 154)	65.6 ± 14.0	50 min	n.a	n.a	Multiple^#^	No improvement of global muscle strength at discharge	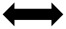	n.a	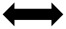	n.a	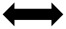
Gruther et al. (2010)	33 (SG = 16, CTRL = 17)	56.0 ± 11.8	30 min (week 1), 60 min (week 2)	n.a	n.a	Multiple^#^	Increase in muscle layer thickness (long-term patient)	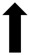	n.a	n.a	n.a	n.a
Meesen et al. (2010)	19 (SG = 7, CTRL* = 12)	67 ± 13.0	30 min	n.a	n.a	Multiple	Reduction of muscle atrophy in stimulated limb	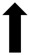	n.a	n.a	n.a	n.a
Dos Santos (2020)	51 (SG = 36, CTRL = 15)	53.2 ± 12.2	55 min	n.a	15.9 ± 3.5	Multiple^#^	Shorter MV duration	n.a	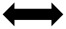	n.a	n.a	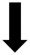
**Stimulated muscle: 2–4 leg muscle groups**												
**Treatment ≤ 10 days**												
Fontes Cerqueira et al. (2018)	59 (SG = 26, CTRL = 33)	42 ± 13.7	60 min	n.a	n.a	Cardiac surgery	No effect on muscle strength, functional independenc, and quality of life	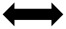	n.a	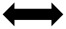	n.a	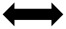
Gerovasili et al. (2009)	26 (SG = 13, CTRL = 13)	57.5 ± 19.7	55 min	9.0 ± 3.1	18.5 ± 4.7	Multiple^#^	Less decrease of CSD of the right rectus femoris and vastus intermedius	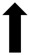	n.a	n.a	n.a	n.a
Waldauf et al. (2021)	150 (SG = 75, CTRL = 75)	61.1 ± 15.3	31.1 min	8.8 ± 2.9	22.2 ± 6.6	Multiple^#^	No difference in physical component summary of SF-36 at 6 months followup	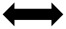	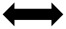	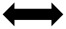	Less negative daily nitrogen balance	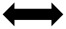
**Treatment > 10 days**												
Falavigna et al. (2014)	11 (SG = 11, CTRL* = 11)	34.0 ± 17.3	20 min	n.a	15.7 ± 4.5	Multiple^#^	No effect on muscle atrophy	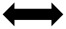	n.a	n.a	n.a	n.a
Kho et al. (2015)	34 (SG = 16, CTRL = 18)	55.1 ± 16.9	60 min	5.9 ± 3.4	25.0 ± 6.9	Multiple^#^	Greater increase in lower extremity strength from awakening to ICU discharge in	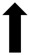	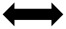	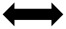	n.a	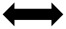
Routsi et al. (2010)	140 (SG = 68, CTRL = 72)	59.5 ± 18.5	55 min	9.0 ± 3.0	18.0 ± 4.5	Multiple^#^	Higher Muscle strength and reduced ICU time	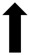	n.a	n.a	n.a	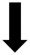
Zanotti et al. (2003)	24 (SG = 12, CTRL = 12)	65.4 ± 6.3	30 min	n.a	n.a	COPD	Improvement of muscle strength and faster ambulation	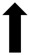	n.a	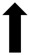	n.a	n.a
**Treatment not specified**												
Parry et al. (2014) ^‡^	16 (SG = 8, CTRL = 8)	61.5 ± 17.6	20–60 min	n.a	20.3 ± 7.4	Sepsis	No effect on Physical Function in Intensive Care Test Score	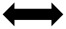	n.a	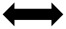	n.a	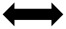
**Stimulated muscles: legs and arm**												
**Treatment ≤ 10 days**												
Nakanishi et al. (2020)	36 (SG = 17, CTRL = 19)	69.3 ± 4.6	30 min	8.2 ± 4.4	24.6 ± 8.7	Multiple^#^	higher muscle layer thickness and cross-sectional area	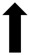	n.a	n.a	n.a	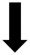
**Treatment > 10 days**												
Akar et al. (2017)	30 (SG = 30)	66.9 ± 13.0	n.a	n.a	n.a	COPD	no difference in pre- and post- manual muscle strength values	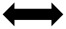	n.a	n.a	CRP and Interleukin-6/-8 levels: no effect	n.a
Rodriguez et al. (2012)	16 (SG = 16, CTRL* = 16)	71.6 ± 13.7	60 min	10.4 ± 2.4	21.8 ± 7.3	Sepsis	Muscle strength and circumference higher on stimulated side	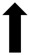	n.a	n.a	n.a	n.a
**Stimulated muscles: 2–4 leg muscles + abdomen**												
**Treatment ≤ 10 days**												
Nakamura et al. (2019)	94 (SG = 47, CTRL = 47)	75.6 ± 12.1	20 min	8.7 ± 3.3	22.9 ± 5.2	Multiple^#^	Reduced muscle volume loss	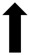	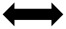	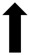	n.a	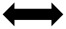
**Stimulated muscles: abdomen and chest**												
**Treatment ≤ 10 days**												
Dall’ Acqua et al. (2017)	25 (SG = 11, CTR = 14)	58.5 ± 14.1	30 min	n.a	27.5 ± 6.2	Multiple^#^	No reduction of muscle thickness	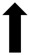	n.a	n.a	n.a	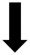
Mc Caughey et al. (2019) ^‡^	20 (SG = 10, CTRL = 10)	58.8 ± 17.6	60 min	n.a	81.8 ± 7.7^§^	Multiple^#^	No effect on muscle thickness	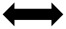	n.a	n.a	n.a	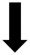

Summary of studies ordered by main modifiers “stimulated muscles” and “overall stimulation duration”, with respect to influence on muscle parameters, disease severity, functional outcomes/ambulation, biomarkers, and duration of mechanical ventilation/ICU, length of stay.

Main outcomes were extracted based on authors’ indication on significant between-group differences. Data on age and disease severity scores is given as mean ± SD. APACHE = Acute Physiology And Chronic Health Evaluation Score (0–71, higher scores indicate more severe conditions), COPD = chronic obstructive pulmonary Disease, CRP = C-reactive protein, CSA = cross sectional area, CSD = cross sectional diameter, CTRL = control group, ICU = intensive care unit, MV = mechanical ventilation, n. a. = not assessed, SG = stimulation group, SOFA = Sepsis-Related Organ Failure Assessment score (0–24, higher scores indicate more severe conditions). Muscular strength/volume/histology/functional independence/ambulation: 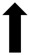
indicates significant improvement, SOFA/APACHE Score/duration of mechanical ventilation/ICU length of stay: 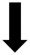
indicates significant reduction, 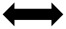
indicates no significant change. * study included interindividual control (one leg EMS, one leg control), ^#^a significant number of patients in the study were diagnosed with sepsis; ^‡^functional electrical stimulation (FES) was used; ^§^ Apache III score.

**TABLE 2:** Summary of EMS stimulation parameters.

References	Stimulation device	Treatment days (d)	Treatment frequency (d)	Sessions (N)	Session duration (min)	Frequency (type of impulse^§^) (Hz)	Pulse width (µs]	Duty cycle (s)	Impulse ramp (on/off^Ɣ^) (s)	Total treatment time (min)	Total treatment time/day (min)	Intensity
Abu-Khaber et al. (2013)	Dr. Eldakr Digital Electronic Acupunctoscope (2D trading company, Hong Kong)	≤28 d	1/d 5 d/wk	N.A.	60 min (5/50/5)	50 Hz (biphasic symmetric)	200 µs	15/N.A.	1 s	-	-	visible or palpable contraction (∼100–150 mA)
Akar et al. (2015)	MI-theta PRO (Compex, Switzerland)	28 d	5 d/wk	20	N.A.	50 Hz (biphasic symmetrical)	600 µs	6/x	1.5/0.75 s	-	-	visible and palpable contraction; max. tolerance: 20–25 mA
Berney et al. (2020)	RT-300 (Restorative Therapies Ltd., Baltimore, United States)/RehabStim FES (Hasomed, Magdeburg, Germany)	11.4 ± 7.5 d	1/d	5.7 ± 4.5	∼60 min	43.5–50 Hz (biphasic)	250 µs/300 µs	N.A.	N.A.	-	-	visible or palpable contraction: ∼ 20–30 mA
Chen et al. (2019)	Omnistm 500 (ZMI, Taipei, Taiwan)	10 d	2/d	20	30 min	50 Hz (biphasic)	400 µs	2/4s	N.A.	200 min	20 min	gradually increase till visible contraction
Dall’ Acqua et al. (2017)	Neurodyn II (Ibramed, São Paulo, Brazil)	7 d	1/d	5 ± 2	30 min	50 Hz	300 µs	3/10 s	1 s	35 min	5 min	visible or palpable contraction; adjusted according to tolerance
Dirks (2015)	TensMed S84 (Enraf-Nonius, Rotterdam, Netherlands)	7.0 ± 1.8 d	2/d	14^#^	40 min (5/30/5)	5/100 Hz (biphasic)	250 µs/400 µs	5/10 s	0.75 s	187 min	27 min	visible and palpable contraction; gradually increase every 3 min
Falavigna et al. (2014)	Dualpex Sport 961 (Quark Medical Products; Piracicaba, São Paulo, Brazil)	10.2 ± 9.0 d	1/d	10	20 min/muscle group	50 Hz (biphasic symmetric)	400 µs	9/9 s	2 s	100 min	10 min	visible or palpable contraction
Fischer et al. (2016)	Compex-3-Pro (Cefar-Compex Medical AB, Malmö, Sweden)	4.9 ± 3.9 d	2/d	10^#^	30 min	66 Hz (biphasic rectangular)	400 µs	3.5/4.5s	0.5 s	131 min	27 min	visible and palpable contraction; right thigh: Ø 40.5 mA left thigh: Ø 40 mA
Fontes Cerqueira et al. (2018)	Neuromed 4082 IFC (Carci, Brazil)	5 d	2/d	9.4 ± 1.6	60 min	50 Hz	400 µs	3/9s	N.A.	141 min	28 min	visible or palpable contraction; M. quadriceps: Ø 54.9 mA. M. gastrocnemius: Ø 49.5 mA
Fossat et al. (2018)	Rehab 400 (Cefar-Compex Medical AB, Malmö, Sweden)	≤28 d	1/d 5 d/wk	N.A.	50 min	45 Hz	400 µs	12/6s	0.8 s	-	-	visible contraction
Gerovasili et al. (2009)	Rehab 4 Pro (Cefar-Compex Medical AB, Malmö, Sweden)	7 d	1/d	7^#^	55 min (5/45/5)	45 Hz (biphasic symmetric)	400 µs	12/6s	N.A.	257 min	37 min	visible or palpable contraction; M. quadriceps: 38 ± 10 mA. M. peroneus longus: 37 ± 11 mA
Gruther et al. (2010)	Compex-Sport-P (Medi-Konzept GmbH, Wiesbaden, Germany)	28 d	5 d/wk	20^#^	30 (wk 1) to 60 min (wk 2–4)	50 Hz (biphasic symmetric)	350 µs	8/24s	N.A.	263 min	9 min	Patient-adjusted max. tolerable muscle contraction
Kho et al. (2015)	CareStim (Care Rehab, McLean, United States)	9.1 ± 8.7 d	1/d	9^#^	53 min (11)	50 Hz (biphasic asymmetric)	Quad.: 400 µs	5/10s	2/<1 s	159 min	17.5 min	visible contraction; gradually increase to maximum
							Tib. ant: 250 µs	5/5s		239 min	26 min	
Koutsioumpa et al. (2018)	EN-STIM 4 (Enraf-Nonius, Rotterdam, Netherlands)	10 d	1/d	10	60 min	50 Hz (biphasic symmetric)	500 µs	N.A.	N.A.	-	-	visible and palpable contraction
McCaughey et al. (2019)	Continuum (Empi, Inc.; Clear Lake, United States)	N.A.	2/d 5 d/wk	12 ^¥^	30 min	30 Hz	350 µs	N.A.	N.A.	-	-	strong visible contraction (Ø 60 mA)
Meesen et al. (2010)	NeuroTrac (Verity Medical Ltd., Hampshire, United Kingdom)	4–22 d	1/d	N.A.	30 min (5/20/5)	5/100 Hz (biphasic symmetric)	250 µs/330 µs	7/14s to 90/30s	2 s/N.A.	-	-	visible and palpable contraction; sub-max. ES: 35–85 mA. daily adjustment: 2–10 mA
Nakamura et al. (2019)	G-TES (Homer Ion Corp, Osaka, Japan)	10 d	1/d	10	20 min	20 Hz	250 µs	5/2s	N.A.	143 min	14 min	adjusted according to patients’ response or expression in vital signs
Nakanishi et al. (2020)	Solius (Minato Medical Science, Osaka, Japan)	5 d	1/d	5	30 min	20 Hz (monophasic rectangular)	650 µs	0.4/0.6s	N.A.	60 min	12 min	visible contraction; M. biceps brachii Ø 30 mA M rectus femoris: Ø 41 mA
Parry et al. (2014)	RT-300 (Restorative Therapies Ltd., Baltimore, United States)	N.A.	5 d/wk	8.6 ± 2.5	20–60 min	30–50 Hz (monophasic rectangular)	300–400 µs	N.A.	N.A.	-	-	visible contraction (max. 140 mA)
Poulsen et al. (2011)	Elpha 3,000 (Danmeter, Odense, Denmark)	7 d	1/d	7	60 min	35 Hz (biphasic)	300 µs	4/6s	0.5 s	168 min	24 min	50% above threshold current for visible contraction; daily adjustment
Rodriguez et al. (2012)	Multiplex Classic (Meditea, Buenos Aires, Argentina)	17.0 ± 18.6 d	2/d	34^#^	30 min	100 Hz (biphasic)	300 µs	2/4s	N.A.	340 min	20 min	gradual increase until visible contraction
Routsi et al. (2010)	Rehab 4 Pro (Cefar-Compex Medical AB, Malmö, Sweden)	14.0 ± 17.6 d	1/d	14^#^	55 min (5/45/5)	45 Hz (biphasic symmetrical)	400 µs	12/6s	0.8 s	513 min	37 min	visible or palpable contraction
Strasser et al. (2009)	Cefar stimulation tool (Cefar-Compex Medical AB, Malmö, Sweden)	4 d	1/d	4	30 min	50 Hz	250 µs	8/4s	N.A.	80 min	20 min	Max. tolerable muscle contraction
Dos Santos et al. (2020)	Sonophasys EUS0503 (KLD Biosistemas, Amparo, Brazil)	7.1 ± 5.3 d	2/d	11.7 ± 9.4	55 min (5/45/5)	45 Hz (biphasic symmetric)	400 µs	12/6s	0.8 s	429 min	60 min	visible or palpable contraction; dosimetry kept constant
Waldauf et al. (2021)	RT-300 (Restorative Therapies Ltd., Baltimore, United States)	6.5 ± 6.1 d	1/d	7^#^	31.1 ± 10.1 min (5/21/5)	40 Hz	250 µs	N.A.	N.A.	-	-	lowest output to allow locomotive movement (range 0–60 mA)
Zanotti et al. (2003)	SportTrainer (Actionfit; Forli, Italy)	28 d	5 d/wk	20	30 min (5/25)	8/35 Hz (biphasic symmetric rectangular)	250 µs/350 µs	N.A.	N.A.	-	-	N.A.

Mean values are given ±SD., total treatment time represents the overall time a current was applied to the stimulated muscle considering treatment and session duration, session length, number of sessions and the duty cycle (on/off time), see methods for detailed description.^§^ as described by authors, not all studies reported the type of impulse; ^Ɣ^ as described by authors, presented as current rise and fall, two values describe alternating settings;^#^ calculated/estimated from reported data of the specified ICU, stay and/or from specified training sessions; ^¥^value calculated from described mean total training time (366 min) divided by duration of one single training session (30 min); N.A., data not available.

### Isolated Stimulation of Quadriceps Muscles

#### Study Characteristics

Twelve studies [46.2%, n = 864 (males = 581, females = 283), see Table 1] limited EMS treatment to the quadriceps muscles (Strasser et al., 2009; Gruther et al., 2010; Meesen et al., 2010; Poulsen et al., 2011; Abu-Khaber et al., 2013; Dirks et al., 2015; Fischer et al., 2016; Fossat et al., 2018; Koutsioumpa et al., 2018; Chen et al., 2019; Berney et al., 2020; Dos Santos et al., 2020). Of these, six reported ≤10 treatment days, and 6 reported >10 treatment days (Table 1). Mean age varied between 53.2 ± 12.8 and 75.7 ± 16.1 years. The applied net EMS treatment time varied between 80 and 429 min. Seven studies included patients with multiple diseases, and four studies included patients with either respiratory failure, septic shock, abdominal surgery, or cardiac surgery. Four studies reported baseline SOFA scores ranging from 7.3 ± 9.4 to 11.4 ± 4.4 points, and seven studies reported baseline APACHE scores ranging from 15.9 ± 8.7 to 29.3 ± 3.7 points (Table 1). Two studies applied functional EMS combined with in-bed cycling and provided additional data on 6 and/or 12-months follow-up (Fossat et al., 2018; Berney et al., 2020).

#### Main Effects

Out of 10 studies investigating muscle parameters, six (60%) reported significantly larger improvement in the EMS group compared to the control group (≤10 days, n = 4; >10 days, n = 2) (Table 1). However, two studies reported contradicting results for different muscular variables. Out of five studies reporting on changes in SOFA/APACHE scores, none detected a significant reduction in patients treated with EMS compared to controls. Seven studies analyzed the duration of mechanical ventilation and/or ICU length of stay of which two (28.6%, treatment >10 days) found a significant reduction in the EMS group compared to the control group. Five studies reported on functional independence and/or ambulation, of which none found a significant difference between groups. One study reported a significantly greater increase of total RNA content from muscle biopsies in the stimulated leg compared to the unstimulated control leg (Strasser et al., 2009).

### Combined Stimulation of two to four Leg Muscle Groups

#### Study Characteristics

Eight studies [30.8%, n = 460 (male = 302, female = 147, not specified = 11), see Table 1] applied EMS to two to four muscle groups including quadriceps, tibialis anterior, gastrocnemius, peroneaus longus, gluteal, and hamstrings muscles in different combinations (Zanotti et al., 2003; Gerovasili et al., 2009; Routsi et al., 2010; Falavigna et al., 2014; Parry et al., 2014; Kho et al., 2015; Fontes Cerqueira et al., 2018; Waldauf et al., 2021). Of these, three reported ≤10 treatment days, four reported >10 treatment days, and one study did not specify overall treatment duration (Table 1). Mean age varied between 34.0 ± 17.3 and 65.4 ± 6.3 years. The applied net EMS treatment time ranged from 141 to 513 min. Five studies included patients with multiple diseases including sepsis, and three studies included patients after either cardiac surgery, sepsis, or COPD. Four studies reported mean baseline SOFA scores ranging from 5.9 ± 3.4 to 9.0 ± 3.1 points. Six studies reported mean baseline APACHE scores ranging from 15.7 ± 4.5 to 25.0 ± 6.9 points. One study applied functional EMS by means of electrical stimulation-assisted cycling (Parry et al., 2014).

#### Main Effects

All eight studies reported on muscle parameters and three (37.5%) detected significant positive EMS effects compared to control. Of these, one study reported ≤10 treatment days, two applied EMS for >10 days. One study (12.5%) reported on SOFA/APACHE scores after study completion, and did not find significant between-group differences during ICU treatment (Kho et al., 2015) Five studies reported on functional independence and/or ambulation, of which only one study (20%) that applied EMS for >10 treatment days, reported significant improvement compared to the control group (Zanotti et al., 2003). One study reported that EMS improved nitrogen balance significantly better compared to control, indicating a reduction in loss of muscle mass (Waldauf et al., 2021). Five studies reported on the influence of EMS on the duration of mechanical ventilation and/or ICU length of stay of which one study (stimulation >10 days) detected a significantly greater reduction in duration of mechanical ventilation compared to control (Routsi et al., 2010).

### Combined Stimulation of Legs and Arms

#### Study Characteristics

Three studies [11.5%, n = 82 (male = 46, female = 36), see Table 1] treated legs and arms including biceps brachii, triceps brachii, wrist flexors and extensors, deltoid, posterior thigh, quadriceps, tibialis anterior, and gastrocnemius muscles (Rodriguez et al., 2012; Akar et al., 2017; Nakanishi et al., 2020). Of these, one study applied EMS for ≤10 treatment days, and two studies for >10 treatment days (Table 1). Age varied between 66.9 ± 12.99 and 71.6 ± 13.7 years. Two studies (66.7%) compared EMS application at one side of the body to the non-stimulated side, the third study used an independent control. The applied net EMS treatment time ranged from 60 to 340 min. One study included patients with multiple diseases including sepsis, one included patients with sepsis only, and one included COPD patients. Two studies reported baseline SOFA scores of 8.2 ± 4.4 and 10.4 ± 2.4 points, and APACHE scores of 21.8 ± 7.3 and 24.6 ± 8.7 points.

#### Main Effects

Three studies of this group reported on muscle parameters, of which two reported significantly greater improvements compared to control, with one study applying EMS for ≤10 days, and two studies applying EMS for >10 days (Table 1). No study reported on changes in SOFA/APACHE scores or on functional independence and/or ambulation. In terms of EMS effects on biomarkers, one study reported a significant reduction of serum CRP and Interleukin-8 levels within the EMS group but did not report on between-group comparison of biomarker changes (Akar et al., 2017). One study reported on the influence of EMS on the duration of mechanical ventilation and/or ICU length of stay and found significantly shorter durations in the EMS treatment group compared to control (Nakanishi et al., 2020).

### 2–4 Leg Muscle Groups and Abdomen

#### Study Characteristics

Only one study [n = 37 (male = 25, female = 12) mean age 75.6 ± 12.1 years, see Table 1] was identified that performed EMS of two to four leg muscle groups plus abdominal stimulation using belt-type electrodes applied to the waist, above the knees, and above the ankles of both sides for >10 days in a cohort with multiple diseases (Nakamura et al., 2019). The study applied a net EMS treatment time of 150 min in patients with a baseline mean SOFA score of 8.7 ± 3.3 points and a baseline mean APACHE score of 22.9 ± 5.2.

#### Main Effects

The study reported significantly less muscle volume loss in the stimulation group, and a significant greater improvement in functional independence/ambulation in terms of stair climbing, without any significant differences in ICU length of stay between the stimulation group and control group (Nakamura et al., 2019).

### Abdomen and Chest

#### Study Characteristics

Two studies (7.7%) treated abdomen and chest muscles [n = 45 (male = 28, female = 17), see Table 1], one for ≤10 and the other for >10 treatment days (Dall’ Acqua et al., 2017; McCaughey et al., 2019). Mean age was 58.8 ± 17.6 and 58.5 ± 14.1 years. The net EMS treatment time was 143 min (only one study provided sufficient data). Both studies included patients with multiple diseases including sepsis, however no study reported a baseline SOFA score. Mean baseline APACHE score was 27.5 ± 6.2 and 81.8 ± 27.7 points (APACHE III). None of the studies reported on 6-months follow-up assessments or long-term effects.

#### Main Effects

Both studies investigated muscle thickness and one detected differential effects in terms of preserved muscle thickness in the EMS group compared to control and reported a significantly larger reduction of ICU length of stay (Table 1). The studies did not investigate changes in SOFA/APACHE scores, functional outcome measures, or biomarkers.

### Safety and Adverse Events

No severe adverse events related to EMS were reported by any of the included studies. One study reported hypotension in two patients and pain in one patient during one EMS session (event rate 1.15%) (Fontes Cerqueira et al., 2018). Berney et al. (2021) reported an adverse event rate of 1.7% (not specified) in the FES group and 3.0% in the control group. Fossat et al. (2018) reported the need for therapeutic intervention in 4.4 and 5.8% in the EMS and control group, respectively with two events that led to a stop of FES. Parry et al. (2014) reported transient blood oxygen desaturation posttraining in one patient. Rodriguez et al. (2012) reported pain in two patients and a case of superficial burn after EMS due to incorrect stimulation settings (event rate 0.7%). Three studies reported no adverse events related to the intervention (Meesen et al., 2009; Gruther et al., 2010; McCoughey et al., 2019). The remaining studies did not report on adverse events or did not specify the number of events related to EMS.

### Risk of Bias

**FIGURE 2:**
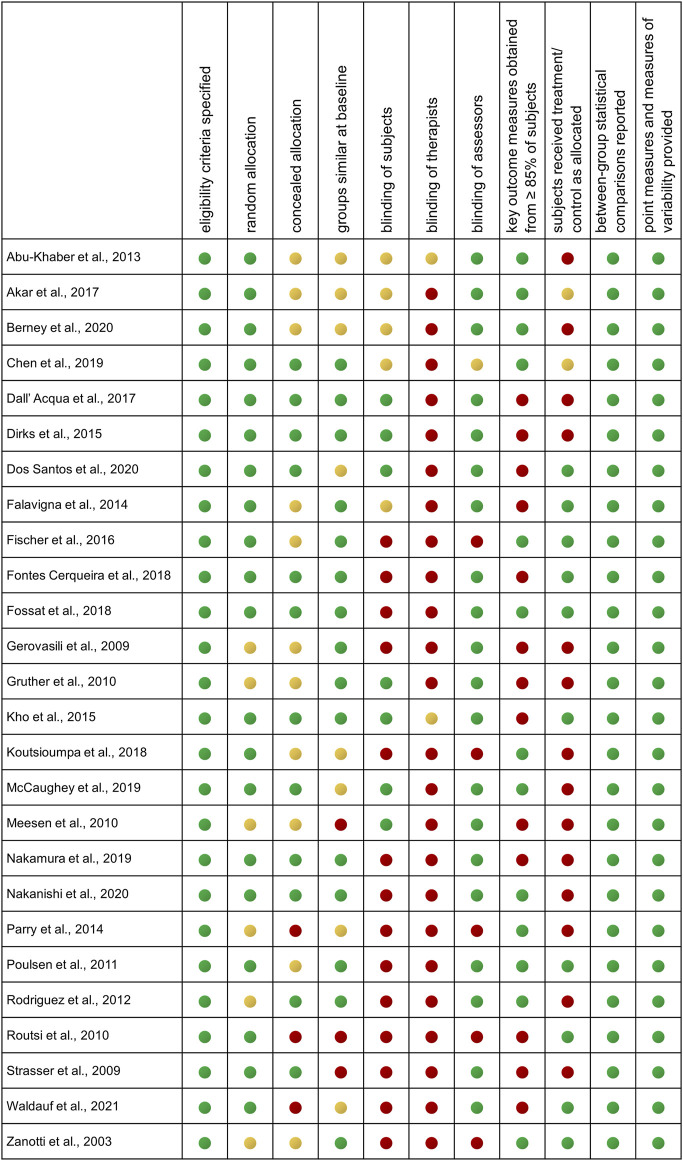
Risk of bias assessment by PEDro scale for individual studies. Authors’ judgement on the methodological quality of each included study assessed by the 11-item PEDro scale. Results are shown for each individual study. Green indicates low risk of bias, yellow indicates unclear risk of bias, and red indicates high risk of bias.

**FIGURE 3:**
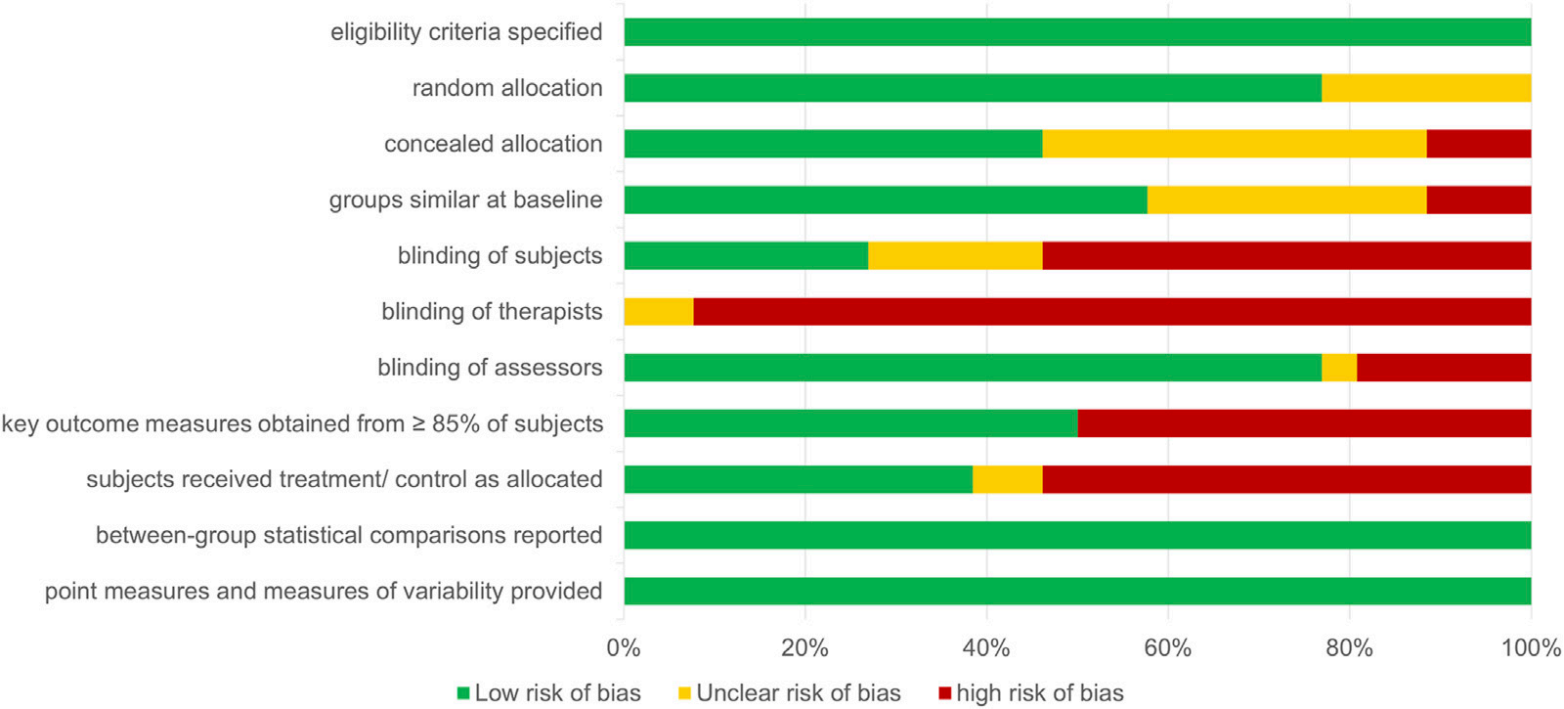
Risk of bias assessment by PEDro item, summary. Summary of authors’ judgement on the methodological quality of included studies assessed by the 11-item PEDro scale. Results are given as percent of studies for each item. Overall, the detected risk of bias was high. Green indicates low risk of bias, yellow indicates unclear risk of bias, and red indicates high risk of bias.

Overall, the risk of bias of the analyzed studies was high (Figures 2, 3). Due to the specific therapeutic treatment under investigation and the standard procedure to adjust individual EMS currents using visible/palpable muscle contractions, considerable deficits in blinding of therapists and subjects (as well as accessors) were present in the majority of studies. More than 50% of included studies did not obtain key outcome measures from ≥85% of subjects, which was partly based on the severity of patients’ conditions on ICU and the associated high mortality rates. In addition, subjects did not receive treatment as allocated in more than 50% of the studies. Even though most studies reported on between-group statistical comparisons, changes over time between groups were not analyzed in a number of studies partly because of missing baseline assessment of variables. In addition, the statistical tests used by some studies to compare groups appeared inappropriate.

### Influence of Stimulation Parameters on Study Outcomes

Studies used a range of different EMS devices with impulse frequencies between 5 and 100 Hz and pulse width between 200 and 650 µs (Table 2). In general, individualized treatment with visible, palpable muscle contractions was used to adjust the applied current (mA), while some studies additionally adjusted to patients’ feedback on tolerance. Sixteen studies (61.5%) provided sufficiently detailed information for the calculation of the net EMS treatment time (Table 2). Based on the sufficiently high number of studies reporting positive effects on muscle outcome variables (n = 10), an estimation of likely effective EMS parameters was performed. The analysis suggested that a mean of 9.6 (min = 5, max = 14) EMS sessions per week, with 39.6 (min = 20, max = 55) minutes per session at a stimulation duration of 5.4 s [45 (min = 25, max = 71)% duty cycle], a stimulation frequency of 50 (min = 20, max = 100) Hz, and an impulse width of 375 (min = 250, max = 650) µs was effective. The effective mean net EMS treatment time per day was thus approximately 25 min based on the calculation (9.6 × 39.6 × 0.45)/7.

### Influence of Sepsis on Study Outcomes

Nineteen studies (73.1%) included patients with sepsis as primary or secondary diagnosis of which 15 investigated muscle parameters and eight (53.3%) reported significantly larger effects compared to controls (Table 1). Seven studies with septic patients reported on post-intervention disease severity scores (SOFA/APACHE), of which none found significantly greater improvements of scores with EMS treatment compared to controls. Seven studies with septic patients reported on functional outcomes/independence, of which one (14.3%) presented significantly greater improvement for the EMS group compared to controls (Nakamura et al., 2019). Fourteen studies with septic patients reported on duration of mechanical ventilaition/ICU length of stay, of which six (42.9%) suggested a significantly greater reduction. Of the three studies reporting on biomarkers, one included septic patients and reported a significanlty reduced negative daily nitrogen balance in the stimulation group (Waldauf et al., 2021).

### Influence of Age and Sex on Study Outcomes

We found a wide age range in the analyzed studies, including patients from 34.0 ± 17.3 to 75.7 ± 16.1 years of age. While older age could be a major modifier with respect to negative study outcomes, no trend for improved outcomes in younger petients for any outcome variable was identified. Although an imbalance of sex distribuation was seen with men being overrepresented in the analyzed populations, four studies included an equal allocation of men and women, of which two (n = 39) presented significantyl better results in muscle parameters compared to controls (Dirks et al., 2015; Chen et al., 2019), while two (n = 46) did not find any significant differences in muscular parameters (Parry et al., 2014; Akar et al., 2017).

### Result Summary

Our investigation indicates large heterogeneity between studies investigating the effects of EMS in critically ill patients on ICU. No pattern in muscular outcomes, post-intervention disease severity (SOFA/APACHE score), functional independence, or ICU length of stay/ventilation with respect to stimulated muscle groups or overall stimulation duration in days was identified. While the effect of sepsis on muscle parameters appears unclear, the effect of sepsis on functional outcomes/independence and duration of mechanical ventilation/ICU length of stay is likely negative and may prevent beneficial responses to EMS. No indications for a modifying effect of age and sex were detected, even though female patients were underrepresented in the analyzed studies.

## Discussion

While a number of studies on most effective EMS parameters, training setup, and moderating conditions on strength and endurance outcomes have been performed in healthy adults and athletes, the knowledge gained is hardly transferrable to optimal EMS application in the critically ill. In this specific patient population, EMS aims to maintain (or reduce the loss of) muscle function and strength and to support early mobilization after critical illness. Due to promising results of individuals studies, EMS has recently also been suggested as an ICU treatment option for COVID-19 patients on ICU (Burgess et al., 2021). Although there has been a rapid increase of studies investigating the implementation of EMS in ICU-acquired weakness, no general recommendation of EMS in the critically ill has been reached. Thus, this work aimed to systematically review the therapeutic evidence on EMS applications in critical illness on ICUs and to provide a qualitative summary of results based on main modifiers. For the first time, an approach to calculate the delivered net EMS treatment time has been included, which may help to streamline comparability of future studies in the field.

So far, two meta-analyses have analyzed the effects of EMS in the critically ill. Recently (Zayed et al., 2020), focused their systematic investigation on EMS effects in RCTs on changes in the Medical Research Council (MRC) grading system as a unified outcome assessment of muscle strength or ICU mortality, MV duration and ICU length of stay and identifies 6 eligible studies (published until November 2018) on patients with any medical or surgical condition (n = 718). Results suggested that EMS combined with usual care did not provide significantly greater improvement of any outcome variable analyzed. The authors concluded that the heterogenous stimulation sites and the primary outcome of MRC measure might have affected this result (Zayed et al., 2020). The study was preceded by a systematic review and qualitative analysis including 12 studies investigating EMS effects using all domains of the International Classification of Functioning, Disability and Health (ICF) framework (Burke et al., 2016). The authors reported a high risk of bias of included studies but suggested that EMS may potentially preserve muscle mass and joint range of motion, improve ventilation outcomes, and reduce activity limitations in critical care. With regard to muscle atrophy, the authors noted large heterogeneity of the applied methods and analyzed variables including ultrasound for muscle layer thickness as well as computed tomography for determination of the cross-sectional area and different approaches to measure muscle circumference manually. They performed a quantitative analysis of three studies uniformly using the MRC score to access muscle strength in a RCT design (n = 146), which suggested significantly increased muscle strength with EMS compared to control.

In the present systematic review including 26 studies and 1,312 patients, we used ordering by main modifiers “muscle groups” and “treatment duration” for a qualitative analysis to overcome the considerably large level of heterogeneity in the field. In addition to muscle strength, functional abilities, ventilation outcomes, and ICU length of stay, our analysis included study outcomes of muscle histology and related biomarkers, which may indicate early effect of EMS on cells and tissues, potentially preceding structural and functional muscular changes. This is of relevance, since the observed median treatment duration of 10 days of the included studies indicates an overall short treatment time on ICU.

### Main Modifiers “Stimulated Muscle Groups” and “Treatment Duration”

No common pattern between the type or groups of muscles stimulated and analyzed outcomes in general or within the individual outcome categories was observed. The largest group of identified studies focused on stimulation of the quadriceps muscle (n = 12, ∼45%), since it is the largest muscle group in the human body and important for all weight-bearing activities and thus early functional independence and ambulation (Parry et al., 2018). However, only 60% of studies on quadriceps stimulation reported significantly larger improvement of muscle parameters in the EMS group and out of five studies reporting on functional independence and/or ambulation, none found significant effects. The second largest group of studies investigating stimulation of two to four leg muscle groups also did not show a clear trend to better improvements with EMS for any analyzed outcome domain. All other categories with different stimulated muscle groups comprised only three or less individual studies and were thus too small to draw any conclusion. This also applied to the category “legs and arms” in which two of three studies investigating muscular improvements reported positive results and additional studies in this segment are needed.

**FIGURE 4:**
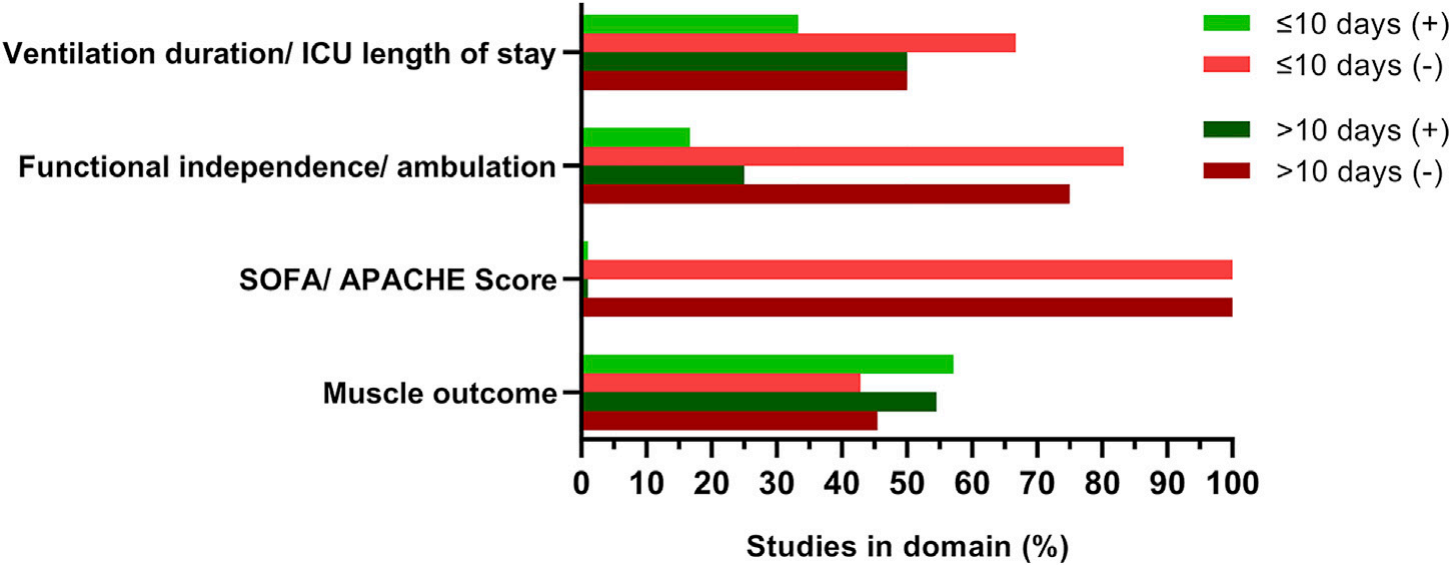
EMS treatment duration did not affect study outcome. Studies were grouped by treatment duration of ≤/> 10 days, respective domain investigated, and reported outcome as presented in Table 1. Green (+) indicates significant positive effects of EMS intervention, red (-) indicates no EMS effects compared to control. Darker colors represent studies with treatment durations >10 days. Percent refers to number of studies investigating EMS effects on outcome variables in the respective domain. Multiple outcomes per study were considered if applicable. No study reported significant positive effects of EMS on disease severity scores [SOFA (Sepsis Related Organ Failure Assessment) and/or APACHE (Acute Physiology And Chronic Health Evaluation)]. ICU, intensive care unit.

The structured analysis of studies that applied EMS for periods longer or shorter than the median treatment duration of 10 days did not reveal any effect of EMS treatment duration in any outcome category investigated Figure 4. Of note, the ratio of studies reporting no effects to studies reporting positive effects of EMS was comparable between studies with shorter and longer treatment duration. This observation may be based on the different study protocols and stimulation parameters applied, including the total number of sessions during the treatment period, session duration and the applied overall contraction time defined by the selected duty cycle. We have thus introduced the concept of calculating the overall applied net EMS treatment time and found that only ∼60% of studies provided sufficient details for this calculation. It is thus highly recommended to report the net EMS treatment time or present data necessary to calculate this variable as the actual or planned treatment duration is far less informative.

### Stimulation Parameters and EMS Protocols

Using a systematic analysis of EMS studies in healthy individuals, it has been suggested that EMS device settings and parameters determine the efficacy of the treatment and the data-derived practical recommendation proposes that 3 EMS sessions per week, at 10–15 min per session for 4–6 weeks may induce strength adaptations in healthy individuals if performed with a duty cycle of 20–25% (3–10 s contraction time), at an intensity of ≥50 mA, with a stimulation frequency of ∼75 (range of 50–100) Hz, and an impulse width of ∼300 (range of 200–400) µs (Filipovic et al., 2011). Since the analyzed studies investigated EMS on top of strength training, the results may however be limited in comparability to ICU patients. In this regard, no study that applied EMS on top of a rehabilitation program in terms of FES reported additional effects on muscle parameters, in line with reports suggesting that neuromuscular adaptations by EMS in disused muscles may rarely be greater than those induced by voluntary contractions (Paillard, 2008).

While the applied impulse width of the studies included in our series equaled the recommended range of 200–400 µs in almost all cases (only three studies applied larger widths), the observed stimulation frequency was considerably lower in the range of 30–50 Hz. Even if it has been argued that EMS (delivered by surface electrodes) does not change the order of physiological muscle fiber recruitment, and that EMS-induced fatigue is not due to preferential recruitment of fast type 2, fatigable fibers, fatigue during EMS needs to be considered. The observed frequencies of 30–50 Hz exceed the firing frequencies of ∼10 and 30 Hz of slow and fast skeletal muscles during voluntary contraction and EMS is accompanied by fixed recruitment of muscle fibers which does not allow alternate recruitment patterns or activation of additional motor units as under voluntary contractions (Gregory and Bickel, 2005). In this regard, it has been shown that higher EMS frequencies (>80 Hz) may lead to increased fatigue with time, while fatigue could be reduced and force levels may be maintained at 20–25 Hz, potentially due reduced excitability of the muscle fiber membrane at higher frequencies limiting restoration of the normal extracellular ionic concentrations (Jones et al., 1979; Dreibati et al., 2010). Of note, among the included studies reporting on beneficial muscular changes with EMS, ∼70.0% applied frequencies of 20–50 Hz, which may induce less fatigue also with regard to the observed mean duration of EMS application of ∼40 min.

In terms of EMS intensity, most studies (76.9%) reported the use of visible muscle contraction as sign of sufficient stimulation intensity and individual feedback on perceived intensity, primarily at the beginning of EMS treatment on ICU, is often not possible. However, there is evidence that critically ill patients may need individually tailored stimulation parameters since the high amount of fluid influx, sepsis, and medication including vasopressors might interfere with electric signal transfer (Segers et al., 2014). Accordingly, the group of Wollersheim categorized ICU patients by EMS response and found that patients with a contractile response at lower electrical currents had lower SOFA scores (Grunow et al., 2019). Excitability at lower intensities may thus be a beneficial precondition for EMS therapy and neurological pathologies and potentially CIP may constitute interfering conditions. In line, Leite et al. (2018) described significant improvements in muscular function, ambulation, and ICU length of stay primarily in non-neurologic cases.

Even though EMS at standard application frequencies induces the non-selective recruitment of all muscle fibers (both types I and II), differences in stimulated muscle groups have been reported. Grunow et al. (2019) suggested, that at a frequency of 50 Hz and a current of up to 70 mA, the mean relative contractile response of the upper extremities of ICU patients was between approximately 50 and 100%, while the mean relative contractile response of the lower extremities was reduced with approximately 0–50%. This appears in line with the observation that leg muscles are more prone to early disuse atrophy than muscles of the upper limbs (Turton et al., 2016). Of note, and despite the fact that the relative distribution of muscle fiber types in arms and legs is comparable, differences in terms of arm and leg muscle glycogen use and subsequent lactate production during exercise have been reported (Helge, 2010) and should be considered for further research and when designing WB-EMS protocols for ICU patients.

Our analysis revealed a low number of adverse events related to EMS, none of which was severe. However, side effects of EMS need to be carefully considered including the potential of EMS to induce rhabdomyolysis. To this respect, a detailed investigation on the safety of EMS in obese sarcopenic men aged 70 years and older did not report adverse events or signs of rhabdomyolysis. In addition, kidney function and N-terminal prohormone of brain natriuretic peptide (NT-proBNP) levels were not affected (Kemmler et al., 2020). A systematic review on the safety of EMS applications reported no adverse or unintended side effect of (WB-) EMS applications but identified several studies with (WB-)EMS-induced CK-levels indicating moderate to severe rhabdomyolysis (Kemmler et al., 2018). Of note, elevated CK-levels appeared related to inappropriately (excessively) high intensity during the initial training sessions, suggesting reduced intensity or treatment time during first EMS application.

### Applied Outcome Measures

With regard to EMS-induced effects on the musculature, muscle strength measurement by MRC classification was a widely used procedure, even though the applicability in the ICU setting with daily variability of patient’s consciousness and cooperation is limited (Hermans and Van den Berghe, 2015). Several studies applied the MRC score at ICU discharge as primary outcome measure without determination of the score at baseline. This procedure should be seen as problematic, not only for the MRC score but for all variables with longitudinal changes, since even in a perfectly randomized study with no significant baseline differences individual changes need to be calculated using adequate statistical methods (Din et al., 2017; Chen, 2017). In general, more objective outcome variables of muscle strength and function are desirable. Only one study used a hand held dynamometer for muscle strength measurement objectivation (Berney et al., 2020). In the population of interest, determination of muscle circumference at baseline and follow-up might be applicable if standardized, also under cost and time efficiency considerations, even though results are so far inconsistent (Fischer et al., 2016; Nakamura et al., 2019; Nakanishi et al., 2020). Some studies applied different imaging techniques to assess EMS-induced changes in the analyzed muscle diameter. While CT scanning and MRI provide reliable information with the highest level of accuracy and reproducibility, there use is likely limited for ICU patients. A more practicable solution for the assessment of skeletal muscle mass may lie in high-resolution ultrasonography, which today represents a valid and reliable tool for providing qualitative and quantitative details including muscle characteristics by cross-sectional area, muscle layer thickness and the pennation angle [see (Formenti et al., 2019) for comprehensive review].

Of the overall 11 studies that evaluated EMS effects on functional independence and ambulation, only 2 studies which used simple mobility scores based on leg muscle strength and function such as transfer from bed to chair, overall mobility, or stair climbing reported significant differences (Zanotti et al., 2003; Nakamura et al., 2019). One reason for the observed negative outcomes of functional independence and ambulation might be, that the applied scales or tests are not appropriate to measure effects of the applied EMS protocols. The Barthel-Index and Functional Independence Measure (FIM), which are frequently used indices of abilities in rehabilitation settings, include three (Barthel Index) and two (FIM) broad items of mobility and personal hygiene, which may not be adequate to detect effects of local EMS stimulation (Fossat et al., 2018; Nakamura et al., 2019). Despite rising evidence for increased excitability of the corticomotor pathway even in response to a single EMS session (Maffiuletti et al., 2018), fundamental multifunctional neurophysiologic improvements including changes of the sensory system, spinal and supraspinal motor tracts, and cognitive improvement necessary for more independence in ambulation (Rothwell, 2012; Prochazka, 2015), are not likely affected by local EMS applied for a treatment duration of 10 days only. Indices used on ICU, such as the short physical performance battery (SPPB), the Physical Function in ICU Test (PFITs), the Functional Status Score of the Intensive Care Unit (FSS-ICU) reflect patients’ mobility and development. However, their validity to detect EMS effects on isolated muscle groups remains a matter of debate even if systemic effects of EMS are assumed. Future studies should thus carefully consider the selection of tests in relation to the stimulated muscle groups and linked physical function.

On the molecular level, EMS has been shown to induce favorable changes in humans and animal models. For example, reduced disuse amyotrophy and bone loss in rats after sciatic neurectomy treated with EMS was accompanied with lower myostatin expression and increased IGF-1 and mechano growth factor (MGF) levels in muscle fibers (Feng et al., 2016). In healthy elderly subjects, myogenic precursor cells showed increased proliferation and gene expression of MYOD/G after EMS and muscle-specific microRNAs (miRs) miR-1, -133, and -206 were upregulated, while enhanced satellite cell fusion with mature skeletal fibers was observed (Di Filippo et al., 2017). Despite these promising findings, only three studies investigated EMS effects on biomarkers in ICU patients with partly positive effects. No study included analysis of serum creatine kinase (CK) despite the known large inter-individual differences in response to EMS (Kemmler et al., 2015).

### Clinical Phenotype, Disease Severity, and Long-Term Follow-Up

The level of CIP may constitute a critical factor for EMS responsiveness (Lim and Han, 2010) since data from animal models suggested that EMS may improve muscle weight, reduce atrophy and markers of apoptosis after partial but not after complete denervation. Diagnostic differentiation of CIP and CIM is possible but requires electrophysiological examinations as one main criterion (Latronico et al., 2012; Mehrholz et al., 2015; Latronico et al., 2017). Since the clinical courses of CIP, CIM, and disuse atrophy are different, the objectivation of underlying neuromuscular disease is crucial to rate and explain EMS effectivity. Even though two studies reported about signs of myopathy after muscle biopsy, none of the included studies performed electrophysiological examinations. Our analysis provides evidence that patients with sever sepsis may not benefit from EMS therapy, which is in line with the findings from previous studies and may be based on the inflammatory and hypercatabolic conditions leading to activation of the ubiquitin proteasome and lysosomal system which, together with high cytokine levels, limit muscle contractility (Rodriguez et al., 2012; Segers et al., 2014; Grunow et al., 2019). Eight studies used SOFA and/or APACHE disease severity scores to determine EMS effects but no study found significant changes in this domain. Again, a number of studies did not evaluate the change from baseline of the respective score, which affects validity of the analysis. While application of disease severity scores is a useful approach to define baseline characteristics of the studied population, some individual items of the SOFA and APACHE scores are unmodifiable by short-term interventions (age, chronic health score), and others may not be affected by EMS or could be masked by short-term EMS effects such as increased leukocyte levels. All studies included in this analysis investigated EMS effects compared to standard care, which was not described in sufficient detail in most studies. To this respect, it is well known that early rehabilitation programs have largely improved in recent years and are implemented in some but not all ICUs (Zang et al., 2020; Dos Santos Moraes et al., 2021). It is thus possible that significant EMS effects were detected in studies performed at locations with less-effective standard care. Berney et al. for example applied EMS in a multicenter setting with well-advanced early rehabilitation programs and did not detect additional improvements with EMS, while reporting greater muscle strength and higher ambulation rates in the control groups compared to other studies. Thus, EMS may not exert additional effects on all ICUs but may be a therapeutic option in settings with certain constrains preventing implementation of ICU rehabilitation programs (Berney et al., 2020). Only two of the included studies presented follow-up data after ICU discharge and no conclusions may be drawn on the long-term effects of EMS. Thus, future studies should try to follow-up on patients to evaluate if EMS provides long-term benefits on frailty and long-time morbidity (Baldwin et al., 2014; Bagshaw et al., 2015).

### Limitations

Some limitations for the presented analysis may exist. First, a high level of heterogeneity was noted and the different clinical populations investigated in combination with the high mortality rates reported in some studies may have affected the overall outcome. Second, the detected risk of bias was high and absence of blinding in particular and in combination with subjective outcome assessment could have affected individual study results. In addition, some studies applied outcome measures (i. e. SOFA/APACHE score) which may not be sensitive to EMS in general. Reporting and publication bias may have affected the present analysis since studies may have remained unreported/unpublished because of negative or non-significant test results. Furthermore, the record search was limited to studies published in English and despite recent suggestions for meta-analysis (Cumpston et al., 2019), grey literature was not included in our analysis. This was done since grey literature may suffer from lower quality checking compared to peer reviewed published material. However, a recent report on the impact of grey literature on results of meta-analyses suggested that inclusion of grey literature rarely impacted the results and conclusions of a review, with larger impact in fields with few available studies (Hartling et al., 2017). Thus, it seems conceivable that our findings and conclusions were unaffected by this limitation.

## Conclusion

Even though the identified studies had a high level of heterogeneity and risk of bias, we conclude that if appropriate protocols are applied, EMS should be applied on top of existing early rehabilitation programs especially when high frequency rehabilitation therapy is either not accessible of not possible due to patients’ condition. The rate of adverse events with EMS appeared equal or lower compared to control interventions and no study reported severe adverse events. Even if some patients may not benefit, EMS offers therapeutic potential for some ICU patients.

### Practical Recommendations

Our findings support the suggestion that EMS treatment should be started as early as possible since muscle fiber degradation and reduction in contractile response proceed from day one on ICU. Individual strong visible and/or palpable muscle contraction should be used to adjust EMS currents daily at an intensity ≥50 mA not exceeding 100 mA. Based on the available data of identified studies reporting positive effects, EMS should be applied daily at a net EMS treatment time of 25 min delivered at a 45% duty cycle during 55 min with a stimulation frequency of 50 Hz and an impulse width of 375 µs. General safety and tolerance criteria should be considered as described (Hodgson et al., 2014). In addition, during the initial EMS session, intensity should be reduced to 40–60% to avoid negative effects such as local irritation/pain and to reduce the potential risk of rhabdomyolysis. In addition, monitoring of creatine kinase (CK) during routine blood work on ICU is suggested to identify early side effects of EMS including rhabdomyolysis. Considering the different response rates at least of upper and lower extremities and the overall likely beneficial systemic effects of EMS, simultaneous stimulation of legs, arms, abdomen and potentially upper and lower back muscles in terms of WB-EMS should be considered using adjustable EMS devices to deliver differential stimulation to respective muscle groups if needed.

Future clinical studies investigating the effects of EMS on critical illness should provide detailed clinical characterization of patients including pre-ICU conditions and neurophysiological examinations for diagnosis of CIM and the level of CIP. Parallel three-arm trials comparing EMS effectiveness in CIP and CIM populations are warranted also to identify EMS non-responders. It is highly recommended to report the net EMS treatment time for better comparability between clinical studies. To individually optimize stimulation parameters and to compensate response variability and fatigue, evoked electromyographically controlled electrical stimulation can be used for monitoring and adjustment of treatment (Hayashibe, 2016). Investigation of EMS-induced changes on the skeletal muscle should preferably be performed using ultrasound examinations at baseline and follow-up. The use of circulating functional biomarkers such as muscle-specific miRNA to monitor and control individual EMS treatment needs further investigation. To document the clinical benefits of EMS, long-term investigations with follow-ups at 6 months after hospital release should be conducted. Future studies should also investigate if combinations of EMS with voluntary contractions, vibration therapy, blood flow restriction, and potentially photobiomodulation could improve (long-term) EMS effects as recently suggested (Blazevich et al., 2021).

## Data Availability

The original contributions presented in the study are included in the article/Supplementary Material, further inquiries can be directed to the corresponding author.
